# GYY4137 a H_2_S donor, attenuates ipsilateral epididymis injury in experimentally varicocele-induced rats via activation of the PI3K/Akt pathway

**DOI:** 10.22038/ijbms.2019.30588.7372

**Published:** 2019-07

**Authors:** Yu-qi Xia, Jin-zhuo Ning, Fan Cheng, Wei-min Yu, Ting Rao, Yuan Ruan, Run Yuan, Yang Du

**Affiliations:** 1 Department of Urology, Renmin Hospital of Wuhan University, Wuhan, Hubei 430060, P.R. China

**Keywords:** Apoptosis, Epididymis, GYY4137, Reactive oxygen species Varicocele

## Abstract

**Objective(s):**

The current study was aimed to investigate the effect of morpholin-4-ium 4 methoxyphenyl (morpholino) phosphinodithioate (GYY4137) on ipsilateral epididymis injury in a rat model of experimental varicocele (VC).

**Materials and Methods:**

Sixty Wistar rats were randomly assigned to sham, sham plus GYY4137, VC and VC plus GYY4137 groups. Sperm quality parameters, including sperm count, motility and viability were evaluated after 4 weeks. Histological changes were measured by hematoxylin and eosin staining between the groups. The oxidative stress levels were estimated by determining epididymal superoxide dismutase (SOD) and malondialdehyde (MDA). The apoptosis status and the expression of phosphatidylinositol 3′-OH kinase (PI3K)/Akt were analyzed by immunohistochemical analysis, western blot and RT-qPCR.

**Results:**

VC resulted in the decrease of sperm parameters, significant histological damage and higher levels of oxidative stress and apoptosis. Compared to the VC group, GYY4137 markedly ameliorated these observed changes. In addition, treatment with GYY4137 obviously reduced the levels of caspase-3 and Bax and increased the levels of the phosphorylation of PI3K p85 and Akt.

**Conclusion:**

Our data demonstrated that GYY4137 may alleviate the sperm damage and epididymis injury in experimentally VC-induced rats by activation of the PI3K/Akt pathway.

## Introduction

Varicocele (VC) is pathological enlargement and tortuosity of the pampiniform venous plexus, which results in progressive genitourinary system damage and infertility (1). Several mechanisms are considered to be associated with the pathogenesis of VC impairing spermatogenesis, including hyperthermia, autoimmunity, hypoxia, endocrine disorder and epididymis disturbance (2, 3). The epididymis that is defined as a structure connecting testicles to the vas deferens preforms an essential part in sperm maturation, sperm concentration and transport (4). Epididymis dysfunction that has been previously overlooked is also related to oxidative stress and apoptosis and causes the reduction of fertilization capacity in men during VC (5). Currently, there are two treatment options for VC repair, including varicocelectomy and percutaneous embolization. Both procedures have been shown to lead to significant improvement in fertility with low recurrence and complication rates (6). However, over 60% of the couples with infertility and VC do not have spontaneous pregnancy within a year after varicocelectomy, and one factor contributing to this outcome may be epididymal damage (7). Therefore, a supplementary therapy for protecting the epididymis during management of VC is required.

Hydrogen sulfide (H_2_S), a poison gas under most conditions, has currently attracted considerable interest as an endogenous gaseous transmitter that elicits cytoprotective effects in mammalian species (8, 9). Decreased H_2_S is considered to be involved in the pathophysiological mechanism of myocardial infarction, septic shock, intestinal barrier injury and chronic renal failure (10-12). Thus, the pharmacological roles of H_2_S donors in several animal studies have been widely reported (13, 14). The morpholin-4-ium 4 methoxyphenyl (morpholino) phosphinodithioate (GYY4137) is a new synthetic H_2_S donor. Compared to conventional inorganic generators, such as Na_2_S and NaHS, GYY4137 generates more stable H_2_S and has a slower production rate, which is similar to the endogenous production of H_2_S (15). Previous studies have shown that GYY4137 protects against tissue injury in cardiovascular, digestive and urinary systems by inhibiting oxidative stress and attenuation of apoptosis in rat models (13, 16-18). However, whether GYY4137 affects VC-induced epididymis injury *in vivo* along with possible signaling mechanisms is unknown.

In the present study, we applied GYY4137 to evaluate the protective effects of H_2_S on epididymis injuries induced by VC in rats. The potential mechanism and intracellular signaling pathways were also investigated.

## Materials and Methods

***Animals***

Sixty adult male Wistar rats (210-250 g) that aged from 8-10 weeks were received from the Hubei Center for Disease Control (Hubei, China). The animals were acclimatized in the animal house of institution at a steady temperature (22 ± 2 °C) and humidity (40-70%) on a 12/12-hr light-dark cycle with free access to water and a small animal diet before the experiment. All handlings were approved by the Animal Experimental Ethics Committee of Wuhan University (Wuhan, Hubei, China). The animal experiments were carried out according to the Guide for the Care and Use of Laboratory Animals (19). 

***Experimental groups and surgical procedures***

According to a previously published method (20), animals were anesthetized with 2% sodium phenobarbital given IP (50 mg/kg; Beijing Propbs Biotechnology. Co., Ltd.) to maintain surgical anesthesia throughout the procedure. After an abdominal median incision, the viscera around the left kidney were removed, and the left renal, adrenal gland, and internal spermatic veins were exposed. The renal vein was elaborately isolated from the junction of renal vein and inferior vena cava, and then a 0.5-mm smooth metallic rod was put parallel below to the left renal vein. Subsequently, 4-0 silk suture was tied to block the left renal vein, which reduced vein diameter by half. After the metal rod was pulled out, the left renal vein dilated within the limits of the suture. Finally, 4-0 silk was used to suture the abdominal median incision. Four weeks later, the spermatic vein and left renal vein exhibited abnormal expansion, elongation and tortuosity, and there were no pathological features in the left kidney, such as atrophy. The sixty rats were stochastically assigned into four groups. In the sham group (group A), the above anesthetic and surgical handlings were operated without left renal vein ligation. In the sham+GYY4137 group (group B), the above anesthetic and surgical handlings were operated without left renal vein ligation, and rats received an IP treatment after the procedures once a day. In the VC group (group C), the VC model was established according to the method described above. In the VC+GYY4137 group (group D), 20 mg/kg GYY4137 (Sigma-Aldrich; Merck KGaA, Germany) was administered IP to each rat after the procedures once a day. 

***Preservation of epididymides***

Four weeks after the VC model was successfully established, all left epididymides were removed, dissected to remove associated adipose tissue and fascia attached to the surface of epididymis, and then left to dry on filter paper after washing the specimens with saline. The epididymides were then cut in half, with one half fixed in 4% paraformaldehyde for histological examination. The remaining part was frozen straight away and reserved at -80 °C for later experiment.

***Assessment of epididymal sperm parameters***

The left epididymis caudal was carefully separated under a 20-times magnification microscope. Then, the epididymides were mincing in 5 ml of Ham’s medium to separate the sperm. After that, the medium was incubated at 37 °C for 15 min. Based on the standard procedure of WHO (2010) recommendations (21), the sperm count was performed.

For motility assay, the sperm samples were diluted to 1:8 in Ham’s F10 medium, and 20 μl of sperm sample was examined under an optical microscope by counting over 200 spermatozoa stochastically in 10 fields. Only the motile spermatozoa with forward progression were counted. Finally, motility was calculated based on the following equation: the mean number of motile spermatozoa x 100/total number of spermatozoa.

To determine sperm vitality, the eosin-nigrosin staining was performed. A 40 μl of freshly sperm suspension was mixed with 10 μl of eosin Y. After 2 min incubation, the semen specimen was transferred to a clean slide. The slide was then observed by microscope, and sperms were counted at a magnification of 400X. The neutral spermatozoa were regarded as viable and pink spermatozoa were regarded as dead (22).

***HE staining***

After fixation in 4% paraformaldehyde, epididymides were imbedded in paraffin and were cut into 5-μm slices. After general dewaxing and hydration, the slices were then dyed with hematoxylin and eosin.

***Biochemical evaluation***

Epididymal malondialdehyde (MDA) level and superoxide dismutase (SOD) activity were quantified spectrophotometrically using MDA and SOD Assay kits following the instructions (Nanjing Jiancheng Bioengineering Institute, China). The MDA level was determined according to the absorbance at 532 nm and presented as nmol/mg protein. SOD activity was measured by checking the absorbance at 550 nm and described as U/mg protein. 

***TUNEL assays***

The apoptosis index (AI) of epididymis epithelial cells was detected with the terminal deoxynucleotidyl transferase dUTP nick end labeling (TUNEL) method using a detection kit (Roche, Mannheim, Germany). TUNEL-positive cells presented stained brown in the nucleus. Five fields were randomly selected from each slide. Approximately, 200 epididymis epithelial cells were calculated in each field. The AI was determined as follow: AI = TUNEL-positive cells / total counted cells × 100%.

***Immunohistochemistry***

The protein expression levels of Bax and caspase-3 were analyzed by immunohistochemical staining. Antibodies were bought from Santa Cruz Biotechnology Co. Ltd, including Bax (sc-493, a rabbit polyclonal antibody) and caspase-3 (sc-7148, a rabbit polyclonal antibody). All procedures were conducted according to the manufacturer’s recommendations. By comparing the staining intensity in microscopic examination, the figures were analyzed.

***Western blot***

All epididymal tissue proteins were extracted and then quantified via the bicinchoninic acid assay. In brief, equivalent quantities of protein samples (40 μg/lane) were prepared for gel electrophoresis, separated by electrophoresis on a sodium dodecyl sulfate-polyacrylamide gel (SDS-PAGE), and subsequently transferred to a polyvinylidene difluoride membrane (Millipore, Billerica, MS). The membrane was blocked using 5% non-fat milk in Tris-buffered saline with Tween 20 (TBST) buffer and then treated with the following major antibodies at 4 ^°^C overnight: Bax (1:500 dilution), caspase-3 (1:1000 dilution), phosphatidylinositol 3′-OH kinase (PI3K) p85(1:1000 dilution), phosphorylated PI3K (p-PI3K) p85(1:500 dilution) Akt (1:1000 dilution) and p-AKT (1:1000 dilution). The above mentioned antibodies were obtained from Santa Cruz Biotechnology (Santa Cruz, CA). After rinsing the membranes twice with TBST buffer, the membranes were treated with secondary antibodies for 1 hr. All specific bands were visualized using an ECL system kit (Pierce Biotechnology, Beijing, China). Optical densities were estimated with ImageJ software (NIH, Bethesda, MD, USA).

***Reverse transcriptionpolymerase chain reaction (RT-qPCR)***

Total RNA was extracted from the epididymal tissue samples using TRIzol reagent (Invitrogen Life Technologies, Carlsbad, CA, USA), and the RNA purity was measured using spectrophotometry. First-strand cDNA was synthesized with a cDNA synthesis kit (Promega Corporation, WI, USA) according to the manufacturer’s instructions. Then, the cDNA was amplified by qPCR using an Applied Biosystems SYBR Green mix kit (Applied Biosystems, CA, USA) and the ABI 7900 Real-Time PCR system (Applied Biosystems Life Technologies, Foster City, CA, USA). The primers were designed as follows: Bax forward, 5’-TCACACCGATGTCCTGACGA-3’, and reverse, 5’-CTGTCATGCAGATGGTCATC-3’; caspase-3 forward, 5’-TGGACTGCGGTATTGAGACA-3’, and reverse, 5’-GCGCAAAGTGACTGGATGAA-3’. Glyceraldehyde 3- phosphate dehydrogenase (GAPDH) was served as a housekeeping gene. The data were measured as a proportion of gene mRNA to GAPDH mRNA (forward, 5’-ACAGCAACAGGGTGGTGGAC-3’, and reverse, 5’-TTTG-

AGGGTGCAGCGAACTT-3’). PCR was performed with 40 cycles at 94 ^°^C for 30 sec, 56^ °^C for 30 sec and 72 ^°^C for 25 sec using the ABI 7900 Real-Time PCR system (Applied Biosystems Life Technologies, Foster City, CA, USA). 

***Statistical analysis***

All data were presented as the mean±SD. Statistical analysis was conducted using SPSS version 19.0 (SPSS Inc., Chicago, IL, USA). The means were compared using one-way analysis of variance followed by the Student-Newman-Keuls test for the different groups. A difference was considered significant at *P*<0.05. All experiments were performed at least three times.

## Results

***GYY4137 improves VC-induced sperm quality damage***

In order to analyze the influence of GYY4137 on sperm quality parameters in VC-induced rats, sperm count, motility and viability were evaluated. The sperm count, motility and viability in the rats of the VC group were significantly decreased comparing to Sham groups and Sham plus GYY4137 group. Conversely, treatment with GYY4137 effectively improved all parameters of semen quality in VC plus GYY4137 group (Table 1).

***GYY4137 ameliorates VC-induced histopathological damage***

**Figure 1:**
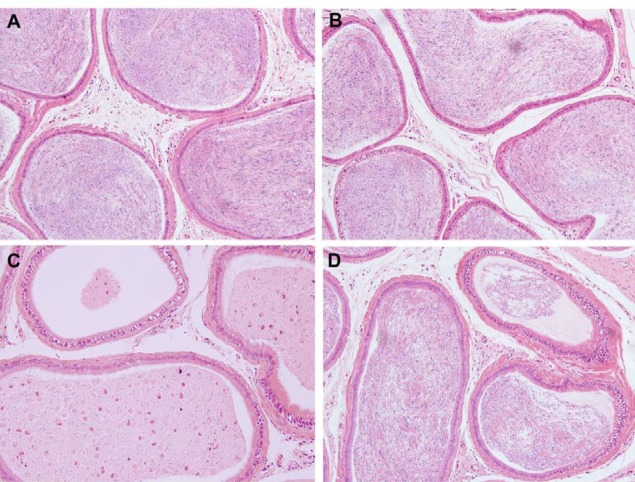
GYY4137 ameliorates varicocele-induced histopathological damage. Epididymis tissues were analyzed using hematoxylin and eosin stain (magnification, x200) in four groups. Groups A and B showed no marked morphological changes. Group C indicated significant pathological and morphological changes. The above changes in the group C were alleviated in Groups D. (A) sham group, (B) sham+GYY4137 group, (C) VC group, (D) VC+GYY4137 group. VC, varicocele; GYY4137, Morpholin-4-ium 4 methoxyphenyl (morpholino) phosphonodithioate

The left epididymal tissue sections were HE-stained and no substantial morphological changes were observed in groups A and B. The ductus epididymis was arranged in an orderly manner and filled with spermatozoa in the lumen. VC resulted in significant histological damage, which was determined by the decreased diameter of ductus epididymis, abnormal aggregation of exfoliated spermatozoa, and widespread damage to the epididymal epithelium. Nevertheless, following treatment with GYY4137, there were fewer histopathological changes in ductus epididymis and epididymal epithelium in group D (Figure 1).

***GYY4137 mediates the decrease in MDA level and increase in SOD activity after VC***

**Figure 2:**
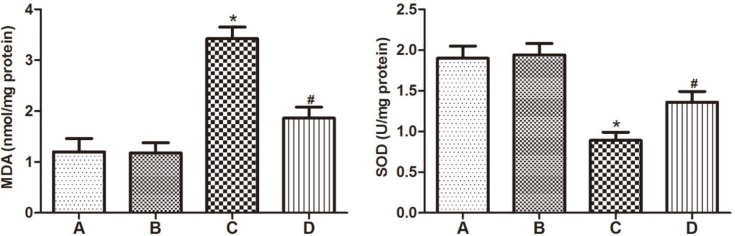
GYY4137 mediates the decrease in MDA level and increase in SOD activity after varicocele. MDA level and SOD activity in the four groups. (A) sham group, (B) sham+GYY4137 group, (C) VC group, (D) VC+GYY4137 group. *P<0.05 vs group A; #*P*<0.05 vs group C. VC, varicocele; GYY4137, Morpholin-4-ium 4 methoxyphenyl (morpholino) phosphonodithioate; MDA, malondialdehyde; SOD, superoxide dismutase

To determine the influence of GYY4137 on biomarkers of oxidative stress status in the epididymis of rats after VC induction, MDA level and SOD activity were detected. The concentration of MDA in the epididymis was notably increased in VC-induced rats in group C compared to groups A and B. In contrast, a significant decrease was observed in the level of SOD activity in group C compared to control animals. Conversely, GYY4137 administration effectively mediated the decrease of MDA level and increase of SOD activity of VC rats in group D (Figure 2). 

***GYY4137 reduced the VC-induced apoptosis of epididymal epithelial cells***

**Figure 3:**
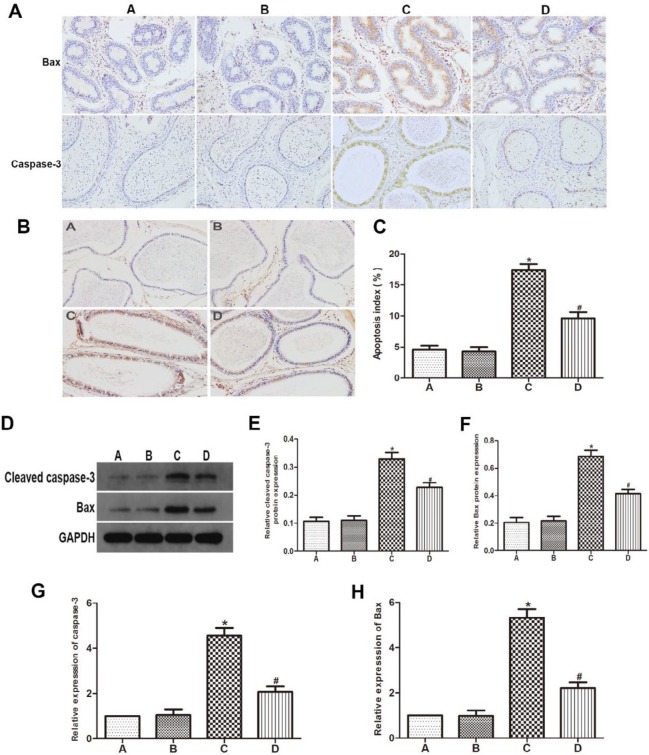
GYY4137 reduced the varicocele-induced apoptosis of epididymal epithelial cells. (A) Immunohistochemistry staining of Bax and caspase-3 in the epididymis; (B,C) Epididymal epithelial cells apoptosis in the four groups; (D-F) Western blot analysis of Bax and cleaved caspase-3 expression; (G,H) RT-qPCR assays were performed to analyze the expression level of Bax and caspase-3. (A) sham group, (B) sham+GYY4137 group, (C) VC group, (D) VC+GYY4137 group. *P<0.05 vs group A; #*P*<0.05 vs group C. VC, varicocele; GYY4137, Morpholin-4-ium 4 methoxyphenyl (morpholino) phosphonodithioate

To estimate the impact of GYY4137 on apoptosis inhibition, AI as well as the levels of Bax and caspase-3 in epididymal epithelial cells were detected by TUNEL, immunohistochemistry, western blot analysis and RT-qPCR. TUNEL staining showed the AI of rats after VC induction in group C was higher compared to control animals. The GYY4137 treatment had a substantially decreased AI compared to group C (Figures 3B, C). Furthermore, the results of immunohistochemistry indicated that rats in group C displayed increased expression levels of Bax and caspase-3 compared to groups A and B, while GYY4137 treatment significantly lowered Bax and caspase-3 releasing in group D (Figure 3A). As expected, western blot analysis and RT-qPCR showed that GYY4137 obviously decreased the protein and mRNA levels of Bax and caspase-3 (Figures 3D-H).

***GYY4137 increased the phosphorylation of PI3K p85 and Akt***

**Figure 4:**
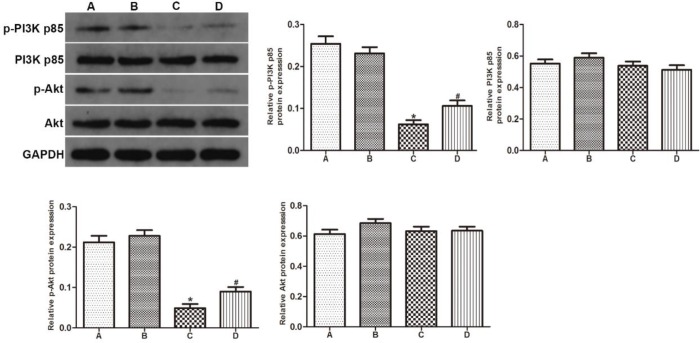
GYY4137 increased the phosphorylation of PI3K p85 and Akt. Protein expression levels of p-PI3K p85, p-Akt, PI3K p85 and Akt were studied by western blot analysis. (A) sham group, (B) sham+GYY4137 group, (C) VC group, (D) VC+GYY4137 group. *P<0.05 vs group A; #*P*<0.05 vs group C. VC, varicocele; GYY4137, Morpholin-4-ium 4 methoxyphenyl (morpholino) phosphonodithioate; PI3K, phosphatidylinositol 3-kinase; p-, phosphorylated

Western blot analysis was performed to investigate the impact of GYY4137 on PI3K/Akt pathway in the epididymides of VC rats. The VC group showed downregulation of p-PI3K p85 and p-Akt in contrast to groups A and B. After treatment with GYY4137, the expression levels of these proteins significantly increased in group D. However, no obvious difference was found in the expression level of total PI3K p85 and total Akt between four groups (Figure 4).

**Table 1:** GYY4137 improves varicocele-induced sperm quality damage

Groups	Sperm count (×million)	Sperm Motility (%)	Sperm Viability (%)
Sham	69.70±2.83	82.7±2.7	90.5±3.4
Sham plus GYY4137	67.20±3.25	83.5±2.3	93.2±2.9
VC	37.34±3.19^*^	46.4±3.1^*^	51.1±2.3^*^
VC plus GYY4137	52.50±2.11^#^	63.1±1.2^#^	70.9±3.9^#^

Values were presented as the mean ± SD. *P<0.05 vs. Sham group; #*P*<0.05 vs. VC group. VC, varicocele; GYY4137, Morpholin-4-ium 4 methoxyphenyl (morpholino) phosphonodithioate

## Discussion

VC is a leading contributing factor for male infertility, and it occurs in approximately 35% of male patients with infertility (23). Although varicocelectomy is considered as the most effective treatment modality for protecting testicular and epididymis function (24, 25), improvement in sperm parameters, including sperm motility, viability and sperm count always requires a significant amount of time, which can be up to 6 months for VC patients (26). Meanwhile, fertility was achieved only in approximately 40% of the patients within a year after surgery (7), which may be the result of changes within the epididymis due to oxidative stress and apoptosis. During transit in the epididymis, spermatozoa acquire their fertilization ability through surface modification of membrane structures and flagellar beating, which is crucial for achieving internal fertilization (4). In this study, VC induced deleterious effects on sperm quality and epididymal morphology, which were consistent with previous results (27). After treatment with GYY4137, we observed an improvement in sperm quality and histological injury of the epididymis compared to the VC rat model.

Oxidative stress that is defined as excess generation of endogenous pro-oxidants or reactive oxygen species (ROS) induces cumulative cellular dysfunction and tissue damage in a variety of disease states (28). In the pathogenesis of VC, the levels of oxidative stress are significantly elevated in the male reproductive tract (29, 30). Excessive ROS leads to cell membrane and nucleus alterations, which result in ultrastructural changes and dysfunction of principal epididymal cells and subsequently leads to infertility (31, 32). MDA is a stabilized end-product of lipid peroxidation. Hence, MDA levels positively correlate with oxidative stress status in semen (33). SOD is classified among the natural antioxidant enzymes that contribute to preventing damage to the cell structure by removing excessive ROS. Therefore, MDA and SOD are often used as biomarkers to estimate the extent of oxidative injury of the epididymis. Consistent with previous studies (34, 35), the significantly higher MDA level and lower SOD activity were observed in epididymides of the VC group in contrast to sham group in our study. Moreover, GYY4137 attenuated VC-induced oxidative stress in rat epididymides. Our investigation demonstrated a protective effect of GYY4137 on epididymides by decreasing the level of MDA and increasing the activity of SOD.

Apoptosis that is defined as programmed cell death performs an essential role by maintaining a normal germ-cell number during spermatogenesis under physiological conditions (36). However, this can lead to infertility under pathological conditions (37). Our previous studies on VC showed that it induced seminiferous epithelial cell apoptosis and spermatogenic cell disorder in testicular tissues of rats (25, 38). According to the results of this study, VC increased the amount of apoptotic epididymal epithelium in contrast to control animals, and apoptosis was significantly improved by GYY4137 according to the TUNEL results. In intrinsic pathway of apoptosis, Bax is an apoptosis-promoting protein among the Bcl-2 family that engages in mitochondrial outer-membrane permeabilization (MOMP) and subsequent cytochrome c (cytc) release (39). Cytc then interacts with downstream caspases, which results in cell death. As one of the apoptosis specific proteinase, caspase-3 is the main factor for apoptosis that initiates morphological and biochemical changes, including cell shrinkage and chromosome fragmentation (40). Numerous anti-apoptosis agents exert a protective effect by influencing the proteins of the mitochondrial pathway (41). To further understand the mechanism of VC-induced apoptosis of epididymal epithelial cells and the protective effect of GYY4137, we examined the changes in apoptosis-related gene expression in the epididymis. We found that ligation of the left renal vein markedly activated Bax and caspase-3, while receiving a treatment with GYY4137 reduced this elevation in apoptosis compared to the VC group. Our data indicated that GYY4137 may attenuate apoptotic processes in VC rat epididymides. 

The PI3K/Akt signaling pathway preforms a significant role as a regulator in the process of cell growth, proliferation and apoptosis (42). PI3K contains two subunits: a catalytic subunit named p110 and a regulatory subunit named p85, which activates Akt through phosphorylation (43). Activated Akt phosphorylates various downstream substrates, including nuclear factor kappa B (NF-κB), Bcl-2-associated death promoter (BAD), and caspase (44). Therefore, many targeted agents activate the PI3K/Akt pathway as a potential therapeutic approach in various disease models. Furthermore, PI3K/Akt pathway was reported to mediate inhibition of apoptosis and protective effects in testicular and epididymal tissues (45, 46). Previous studies have suggested that H_2_S could induce activation of PI3K/Akt pathway to protect against mitochondrial injury (47). Also, Hu *et al*. found that the H_2_S alleviated cardiac dysfunction through reducing apoptosis in impaired PI3K/Akt pathway model by using Akt2-knockout mice (48). Thus, the protective effect of H_2_S would be in correlation with PI3K/Akt pathway. In our studies, we demonstrated that GYY4137 can increase the levels of p-PI3K and AKT, while the total PI3K p85 and Akt were not altered. On the strength of above results, we showed that GYY4137 can reduce cell apoptosis and alleviate epididymis injury through activating the PI3K/Akt pathway.

## Conclusion

In summary, our study provided evidence demonstrating the protective effects of GYY4137 in improving sperm quality, inhibiting the oxidative stress and apoptosis of epididymis through activating the PI3K/Akt signaling pathway in VC-induced rat model of reproductive system damage. Whereas, specific mechanism of GYY4137 in epididymis injury caused by VC remain unknown, and need further investigation.

## References

[1] Damsgaard J, Joensen UN, Carlsen E, Erenpreiss J, Blomberg Jensen M, Matulevicius V (2016). Varicocele Is Associated with Impaired Semen Quality and Reproductive Hormone Levels: A Study of 7035 Healthy Young Men from Six European Countries. Eur Urol.

[2] Sheehan MM, Ramasamy R, Lamb DJ (2014). Molecular mechanisms involved in varicocele-associated infertility. J Assist Reprod Genet.

[3] Zha WL, Yu W, Zhang X, Zheng YQ, Cheng F, Rao T (2011). Effects of artery-ligating and artery-preserving varicocelectomy on ipsilateral epididymis of varicocele-induced rats. Urology.

[4] Sullivan R, Mieusset R (2016). The human epididymis: its function in sperm maturation. Hum Reprod Update.

[5] Vivas-Acevedo G, Lozano-Hernandez R, Camejo MI (2014). Varicocele decreases epididymal neutral alpha-glucosidase and is associated with alteration of nuclear DNA and plasma membrane in spermatozoa. BJU Int.

[6] Johnson D, Sandlow J (2017). Treatment of varicoceles: techniques and outcomes. Fertil Steril.

[7] Abdel-Meguid TA, Al-Sayyad A, Tayib A, Farsi HM (2011). Does varicocele repair improve male infertility? An evidence-based perspective from a randomized, controlled trial. European Urology.

[8] Polhemus DJ, Lefer DJ (2014). Emergence of hydrogen sulfide as an endogenous gaseous signaling molecule in cardiovascular disease. Circ Res.

[9] Wang R (2012). Physiological implications of hydrogen sulfide: a whiff exploration that blossomed. Physiol Rev.

[10] Li L, Whiteman M, Guan YY, Neo KL, Cheng Y, Lee SW (2008). Characterization of a novel, water-soluble hydrogen sulfide-releasing molecule (GYY4137): new insights into the biology of hydrogen sulfide. Circulation.

[11] Nussbaum BL, Vogt J, Wachter U, McCook O, Wepler M, Matallo J (2017). Metabolic, cardiac, and renal effects of the slow hydrogen sulfide-releasing molecule GYY4137 during resuscitated septic shock in swine with Pre-existing coronary artery disease. Shock.

[12] Wu D, Luo N, Wang L, Zhao Z, Bu H, Xu G (2017). Hydrogen sulfide ameliorates chronic renal failure in rats by inhibiting apoptosis and inflammation through ROS/MAPK and NF-κB signaling pathways. Scientific Reports.

[13] Lin S, Visram F, Liu W, Haig A, Jiang J, Mok A (2016). GYY4137, a Slow-Releasing Hydrogen Sulfide Donor, Ameliorates Renal Damage Associated with Chronic Obstructive Uropathy. J Urol.

[14] Lv M, Liu Y, Xiao TH, Jiang W, Lin BW, Zhang XM (2017). GYY4137 stimulates osteoblastic cell proliferation and differentiation via an ERK1/2-dependent anti-oxidant mechanism. Am J Transl Res.

[15] Papapetropoulos A, Whiteman M, Cirino G (2015). Pharmacological tools for hydrogen sulphide research: a brief, introductory guide for beginners. Br J Pharmacol.

[16] Meng G, Zhu J, Xiao Y, Huang Z, Zhang Y, Tang X (2015). Hydrogen sulfide donor GYY4137 protects against myocardial fibrosis. Oxid Med Cell Longev.

[17] Yang F, Zhang L, Gao Z, Sun X, Yu M, Dong S (2017). Exogenous H2S Protects Against Diabetic Cardiomyopathy by Activating Autophagy via the AMPK/mTOR Pathway. Cell Physiol Biochem.

[18] Chen S, Bu D, Ma Y, Zhu J, Sun L, Zuo S (2016). GYY4137 ameliorates intestinal barrier injury in a mouse model of endotoxemia. Biochem Pharmacol.

[19] Laboratory animal welfare (1985). Public Health Service policy on humane care and use of laboratory animals by awardee institutions; notice. Fed Regist.

[20] Turner TT (2001). The study of varicocele through the use of animal models. Hum Reprod Update.

[21] Who WHO (2010). Laboratory manual for the examination and processing of human semen. Int J Androl.

[22] Bjorndahl L, Soderlund I, Kvist U (2003). Evaluation of the one-step eosin-nigrosin staining technique for human sperm vitality assessment. Hum Reprod.

[23] Alsaikhan B, Alrabeeah K, Delouya G, Zini A (2016). Epidemiology of varicocele. Asian J Androl.

[24] Samplaski MK, Lo KC, Grober ED, Zini A, Jarvi KA (2017). Varicocelectomy to “upgrade” semen quality to allow couples to use less invasive forms of assisted reproductive technology. Fertil Steril.

[25] Ning JZ, Rao T, Cheng F, Yu WM, Ruan Y, Yuan R (2017). Effect of varicocelectomy treatment on spermatogenesis and apoptosis via the induction of heat shock protein 70 in varicoceleinduced rats. Mol Med Rep.

[26] Practice Committee of the American Society for Reproductive M, Society for Male R Urology (2014). Report on varicocele and infertility: a committee opinion. Fertil Steril.

[27] Ozturk U, Kefeli M, Asci R, Akpolat I, Buyukalpelli R, Sarikaya S (2008). The effects of experimental left varicocele on the epididymis. Syst Biol Reprod Med.

[28] Sureshbabu A, Ryter SW, Choi ME (2015). Oxidative stress and autophagy: crucial modulators of kidney injury. Redox Biol.

[29] Tawadrous GA, Aziz AA, Mostafa T (2013). Seminal soluble fas relationship with oxidative stress in infertile men with varicocele. Urology.

[30] Blumer CG, Restelli AE, Giudice PT, Soler TB, Fraietta R, Nichi M (2012). Effect of varicocele on sperm function and semen oxidative stress. BJU Int.

[31] Capucho C, Sette R, de Souza Predes F, de Castro Monteiro J, Pigoso AA, Barbieri R (2012). Green Brazilian propolis effects on sperm count and epididymis morphology and oxidative stress. Food Chem Toxicol.

[32] Asadi N, Bahmani M, Kheradmand A, Rafieian-Kopaei M (2017). The impact of oxidative stress on testicular function and the role of antioxidants in improving it: a review. J Clin Diagn Res.

[33] Agarwal A, Virk G, Ong C, du Plessis SS (2014). Effect of oxidative stress on male reproduction. World J Mens Health.

[34] Cho CL, Esteves SC, Agarwal A (2016). Novel insights into the pathophysiology of varicocele and its association with reactive oxygen species and sperm DNA fragmentation. Asian J Androl.

[35] Mostafa T, Rashed LA, Osman I, Marawan M (2015). Seminal plasma oxytocin and oxidative stress levels in infertile men with varicocele. Andrologia.

[36] Hai Y, Hou J, Liu Y, Liu Y, Yang H, Li Z (2014). The roles and regulation of Sertoli cells in fate determinations of spermatogonial stem cells and spermatogenesis. Semin Cell Dev Biol.

[37] Xiao N, Kam C, Shen C, Jin W, Wang J, Lee KM (2009). PICK1 deficiency causes male infertility in mice by disrupting acrosome formation. J Clin Invest.

[38] Zhu SM, Rao T, Yang X, Ning JZ, Yu WM, Ruan Y (2017). Autophagy may play an important role in varicocele. Mol Med Rep.

[39] Chipuk JE, McStay GP, Bharti A, Kuwana T, Clarke CJ, Siskind LJ (2012). Sphingolipid metabolism cooperates with BAK and BAX to promote the mitochondrial pathway of apoptosis. Cell.

[40] Nakagawa A, Shi Y, Kage-Nakadai E, Mitani S, Xue D (2010). Caspase-dependent conversion of Dicer ribonuclease into a death-promoting deoxyribonuclease. Science.

[41] Lin AM, Fang SF, Chao PL, Yang CH (2007). Melatonin attenuates arsenite-induced apoptosis in rat brain: involvement of mitochondrial and endoplasmic reticulum pathways and aggregation of alpha-synuclein. J Pineal Res.

[42] Manning BD, Toker A (2017). AKT/PKB Signaling: Navigating the Network. Cell.

[43] Cantley LC (2002). The phosphoinositide 3-kinase pathway. Science.

[44] Zhao S, Konopleva M, Cabreira-Hansen M, Xie Z, Hu W, Milella M (2004). Inhibition of phosphatidylinositol 3-kinase dephosphorylates BAD and promotes apoptosis in myeloid leukemias. Leukemia.

[45] Al-Maghrebi M, Renno WM (2016). The tACE/angiotensin (1-7)/Mas Axis Protects Against Testicular Ischemia reperfusion injury. Urology.

[46] Dube E, Dufresne J, Chan PT, Cyr DG (2012). Epidermal growth factor regulates connexin 43 in the human epididymis: role of gap junctions in azoospermia. Hum Reprod.

[47] Xu D, Jin H, Wen J, Chen J, Chen D, Cai N (2017). Hydrogen sulfide protects against endoplasmic reticulum stress and mitochondrial injury in nucleus pulposus cells and ameliorates intervertebral disc degeneration. Pharmacol Res.

[48] Hu N, Dong M, Ren J (2014). Hydrogen sulfide alleviates cardiac contractile dysfunction in an Akt2-knockout murine model of insulin resistance: role of mitochondrial injury and apoptosis. Am J Physiol Regul Integr Comp Physiol.

